# Numerical Study on Concrete Pumping Behavior via Local Flow Simulation with Discrete Element Method

**DOI:** 10.3390/ma12091415

**Published:** 2019-04-30

**Authors:** Yijian Zhan, Jian Gong, Yulin Huang, Chong Shi, Zibo Zuo, Yiqun Chen

**Affiliations:** 1 General Engineering Institute of Shanghai Construction Group, Shanghai Construction Group Co., Ltd., Shanghai 200080, China; zuozibo@hotmail.com; 2 Research Institute of Geotechnical Engineering, Hohai University, Nanjing 210098, China; scvictory@hhu.edu.cn; 3 Shanghai Construction Material Co., Ltd., Shanghai 200086, China; 2011cyq0809@tongji.edu.cn

**Keywords:** self-consolidating concrete, pumpability, local flow behavior, discrete element method, parametric study

## Abstract

The use of self-consolidating concrete and advanced pumping system enables efficient construction of super high-rise buildings; however, risks such as clogging or even bursting of pipeline still exist. To better understand the fresh concrete pumping mechanisms in detail, the discrete element method is employed in this paper for the numerical simulation of local pumping problems. By modeling the coarse aggregates as rigid clumps and appropriately defining the contact models, the concrete flow in representative pipeline units is well revealed. Important factors related to the pipe geometry, aggregate geometry and pumping condition were considered during a series of parametric studies. Based on the simulation results, their impact on the local pumping performance is summarized. The present work demonstrates that the discrete element simulation offers a useful way to evaluate the influence of various parameters on the pumpability of fresh concrete.

## 1. Introduction

The growing peak of landmark buildings, such as the Shanghai Tower (632 m high), Burj Khalifa (828 m) and the Jeddah Tower (planned to be over 1000 m), is attributable to the continuous theoretical research and technical development particularly in the field of civil engineering. In high- and super high-rise building projects, concrete pumping has become a very important part of construction technology to guarantee the project schedule. The smooth pumping of fresh concrete directly from the ground surface up to several hundreds of meters height demands an optimal combination of material, equipment and process. The concrete material itself should be highly flowable, which is now generally available thanks to the invention of self-consolidating concrete (SCC) in the late 1980s. The pumping apparatus consists mainly of the pipeline connected to the pump. The pipes should be tough enough to sustain the pressure and abrasion, and the pump should provide sufficient power for the concrete to overcome the gravity and friction. Nevertheless, despite the general success in the completed projects, unexpected situations (for example, inaccurate prediction of pressure, segregation of fresh concrete, leakage of mortar, clogging of aggregates, bursting of pipes, etc.) can still occur in reality, which may significantly influence the project schedule and even cause hazards to the people on site.

Substantial understanding of the flow behavior of fresh concrete during pumping will provide a good foundation for the solution of existing and potential problems in construction projects. Although fresh concrete is relatively less studied as compared to concrete materials and structures in hardened state, scientists and engineers have made considerable efforts in terms of experimental tests, theoretical analyses and numerical simulations. From the mechanical point of view, fresh concrete is nothing but a flowable material similar to smog, plasma, petroleum, debris flow, etc., and can be studied using classical or modern methods for fluids [1].

The flowability, more specifically the rheological property, of fresh concrete is dependent on various factors such as the mix design, internal action and environmental condition, and also complex mechanisms between them [2]. Fresh concrete exhibits non-Newtonian behavior under shear, which can be described by different analytical forms such as Bingham, Herschel-Bulkley or modified Bingham function [3,4]. Standardized experiments such as the slump test are commonly used to assess the workability of fresh concrete owing to its simplicity [5,6,7]. More advanced devices such as rotational rheometer are developed and applied to investigate the material properties and to evaluate the flowability of concrete [8,9,10,11,12]. Full-scale pumping tests are occasionally conducted, which allows directly evaluating the concrete pumping performance [13,14,15,16,17]. In general, experiments are necessary for obtaining the real information concerning the concrete property and the pumping performance; however, experiments can be too expensive and inefficient to investigate a large number of different cases. Based on the experimental data acquired, empirical simplification or theoretical derivation can be considered to quickly estimate the “pumpability” (more specifically, the relation between pumping pressure and pumping rate). The slump value, which is easy to obtain, is used by engineers to assess the pressure loss during pumping [18]. Further research focuses more on the rigorously defined parameters and theoretical models of fresh concrete. For example, in the framework of rheology theory, the yield stress and the viscosity describe the relation between shear stress and shear strain rate; these parameters can be determined from rheometers and introduced into analytical models to evaluate the pumpability [13,15,19,20,21]. In those analytical models, the concrete flow in the pipe is divided into different zones (e.g., the “lubrication layer”, the “shear zone” and the “plug flow”) characterized by different rheological properties. It has been demonstrated that the analytical approach is capable of predicting the pumpability efficiently; however, the simplification of models does not allow reflecting the local complexities of concrete flow. For more detailed analysis of concrete flow behavior, numerical simulation has become a powerful tool.

The rapid development of the numerical simulation of concrete flow in recent years has brought a variety of theory and modeling techniques (see, e.g., [22,23] for an overview). One class of model belongs to the classical computational fluid dynamics (CFD). Typically, the fresh concrete is treated as single phase fluid described by a specific rheological model (such as the Bingham model characterized by a constant viscosity). The Navier-Stokes equations are solved so that the flow of concrete in the problem domain is determined (see, e.g., early work in [24] and recent work in [14]). The CFD is relatively well developed and has been successfully applied in a wide range of research and engineering fields such as aerodynamics, biomedical science, civil engineering and analysis of natural phenomena [25]; however, it is naturally incapable of reflecting the heterogeneity of concrete mixture and to predict complex phenomena such as concrete segregation and pipe blockage.

Another modeling strategy focuses on the explicit representation of concrete heterogeneity. Almost all existing materials (smog, blood, soil, etc.) can be treated as continuum at the macroscopic point of view, because the characteristic length of material ingredient is very small as compared to the problem domain. If one focuses on the local behavior of material at a length scale where the microstructure is no longer negligible, discontinuous modeling should be considered [26]. The computational mechanics for discontinua is a fast-developing field [27]; the discrete element method (DEM) is a typical example of such modeling strategy. The DEM referred to in the present paper can be traced back to the 1970s [28,29]; it was originally proposed for the research of granular assemblies, particularly geomaterials such as rock. Recently, the DEM is also used for the simulation of concrete flow, where the aggregates are represented via particles, and the motion of particles is governed by the Newton’s laws [30,31]. Prior to a DEM simulation, efforts should be made to correlate the mesoscopic modeling parameters and the macroscopic material properties, so that the particle assembly appropriately reflects the real behavior of material. Researchers have also proposed a number of coupled models [32] such as those treating the fresh concrete as fluid containing suspension. The fluid phase represents the mortar and the particle suspension models the aggregates; the flow of mortar is mainly simulated according to CFD and the motion of discrete particles is tracked, with or without further considering the interactions between particles (see, e.g., [33,34,35,36,37]). This way of treating multi-phase material has been also considered in many other topics where the interaction between different phases is significant (see, e.g., the research on gas–solid separation [38], on blood flow [39,40], on fracturing solids [41], etc.) Nevertheless, considering the volume fraction of solid phase in fresh concrete, the accuracy of these methods should be further investigated, since the aggregate packing feature strongly influences the rheological properties of suspension [42].

Concerning the specific concrete pumping problem, few publications report its numerical simulation. As a first choice, CFD is used to simulate the concrete flow as viscous fluid in the pipe; as a result, the total pressure-flow rate relationship is obtained and compared with a real-size pumping experiment [14,43]). In [35], the concrete flow through a bend of pipe is considered, where the DEM is applied in association with CFD in order to investigate the pressure and velocity field and to study the reaction force at pipe wall. As suggested above, CFD is more suitable for the full simulation of pumping under normal conditions, while DEM and coupled methods are more powerful for the analysis of potential hazards during pumping by means of capturing the flow details of aggregate motion.

**Figure 1:**
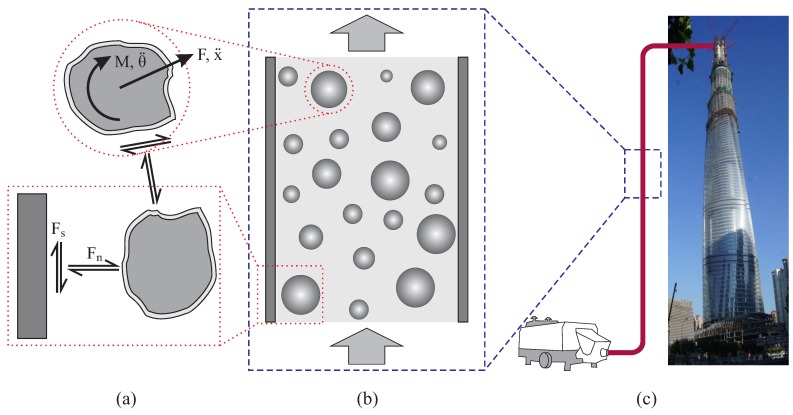
Concept for the concrete pumping analysis: (**a**) material behavior at the mesoscale; (**b**) local concrete flow in pipe; and (**c**) whole system during construction.

This article reports a systematic DEM study of local behavior of fresh concrete during pumping (Figure 1). We investigate the impact of several different factors on the local pumping performance. The main part of paper consists of an introduction of the fundamentals of DEM, the details of modeling and the numerical simulation of representative cases.

## 2. Fundamentals of Discrete Element Method

The *discrete element method* specifically employed in this paper was initially referred to as the *distinct element method* and was proposed for the study of granular assemblies [28,29]. In the past decades, the DEM has gained much attention; it has been considerably enriched for the analyses of various granular materials subjected to discontinuous deformation or motion, such as collision of wet agglomerates, shear bands in the microstructure of materials, flow of irregular particles, fracture in cementitious materials and structures, deformation of underground space, natural hazard of land slide, etc. (see, e.g., [44,45,46,47,48,49,50,51]).

The DEM basically describes the motion of *particles* (Figure 1a). The particle motion is controlled by the Newton’s second law:

(1) $$m\ddot{x}=F,\;I\ddot{\theta }=M$$

where $\ddot{x}$ and $\ddot{\theta }$ stand, respectively, for the second-order time derivatives of position x and orientation θ vectors of a specific particle (i.e., the translational and angular acceleration); *m* and *I* represent its mass and moment of inertia; and F and M are the total force and moment vectors resulted from the constitutive behavior at *contacts* and external body force such as gravity.

The translational and angular velocity ($\dot{x}$ and $\dot{\theta }$) are computed based on the integration of acceleration, following a time-centered integration scheme:

(2) $${\dot{x}}_{\left(n+1/2\right)}={\dot{x}}_{\left(n-1/2\right)}+{\ddot{x}}_{\left(n\right)}\Delta t,\;{\dot{\theta }}_{\left(n+1/2\right)}={\dot{\theta }}_{\left(n-1/2\right)}+{\ddot{\theta }}_{\left(n\right)}\Delta t$$

The position and orientation are updated as

(3) $$x_{\left(n+1\right)}=x_{\left(n\right)}+{\dot{x}}_{\left(n+1/2\right)}\Delta t,\;\theta_{\left(n+1\right)}=\theta_{\left(n\right)}+{\dot{\theta }}_{\left(n+1/2\right)}\Delta t$$

where Δt is the time step size; and the subscripts (n−1/2), (n), (n+1/2) and (n+1) refer to the values at time $t_{n}-\Delta t/2$, $t_{n}$, $t_{n}+\Delta t/2$ and $t_{n}+\Delta t$, respectively. This explicit feature allows DEM to simulate complex nonlinear behavior of granular assemblies with limited use of computer memory; however, a small time step is required, which should be considered as a limitation while using DEM in large scale simulations.

The obtained new position and velocity of particles are used to determine the spatial relations among different contact entities (i.e., particles and boundaries) and to calculate contact forces $F_{c}$ according to the constitutive laws defined for the contacts:

(4) $$F_{c}=F_{c}\left(g,\dot{g}\right)$$

where g and $\dot{g}$ are the *gap* and its time derivative at a specific contact. This equation implies that, in general, the reaction force at contact is dependent on the contact gap (e.g., a spring), and can also be influenced by the changing rate of gap (e.g., a dashpot). A contact model consisting of the normal and shear components can be used for both the particle-particle and particle-boundary contacts (Figure 1a). In the literature, a variety of contact models can be found.

## 3. Modeling of Fresh Concrete with DEM

### 3.1. Geometrical Representation

**Figure 2:**
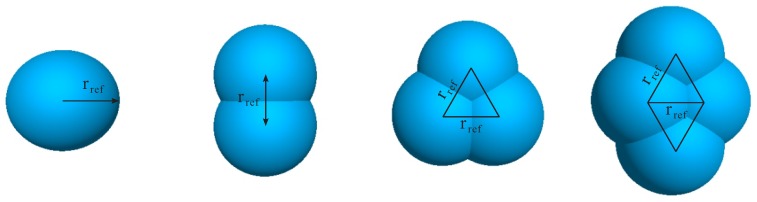
Clump templates used to model concrete aggregates.

Concrete is a heterogeneous material composed of coarse aggregates, fine aggregates, dry cement, water and other chemical additives. In a DEM simulation of concrete flow, the goal is to capture the motion of aggregates that directly reflects the flow behavior of fresh concrete. In the DEM model, an individual particle represents a specific piece of aggregate. Usually in the model, a particle is simply a ball (a disc in 2D), which allows efficiently determining the geometrical relations between different entities during computation. If more attention is paid to the influence of irregular shape of aggregate, non-spherical particles can be generated via specific algorithms (e.g., [52,53,54,55]), and used to investigate different problems related to the shape of aggregate (such as the passing ability of concrete through narrow spaces [31,56]). In the present paper, a compromise has been made between the efficiency and accuracy. The irregular aggregates are represented via manually created clump templates (Figure 2). A clump is a group of rigidly inter-connected overlapping balls. It is believed that using finely modeled clumps better captures the realistic aggregate shapes and should lead to more accurate simulation results; nevertheless, such high resolution of particles (one clump contains typically hundreds of overlapping balls with the size in the order of $10^{-3}$ m) can cause significant increase of computational load. The adopted resolution that a clump consists of only several balls with the size in the order of $10^{-2}$ m already allows reflecting the effect of shape irregularity. Of course, it would be meaningful in the future to conduct experiments and to investigate the influence of particle resolution on the simulation accuracy. While modeling the fresh concrete, every template is used according to a given frequency (for example, as a reference configuration, all four templates are called equally). The size of generated aggregates falls within the given range of aggregate size (e.g., with diameter $d^{\mathrm{agg}}$ = 5–20 mm) either according to a statistical distribution or following a given granulometric curve.

The boundaries (such as container, pipeline and blender), which are already or potentially in contact with the fresh concrete, are usually modeled as rigid walls, either stationary or kinetic according to the given boundary conditions.

### 3.2. Material Model

Fresh concrete is often treated as single phase non-Newtonian fluid from the macroscopic point of view. In the framework of rheology theory, concrete material parameters such as the yield stress and plastic viscosity can be determined based on specific laboratory tests. These parameters are used as input for a typical CFD simulation of concrete flow [8,11,22,57].

In DEM, the bulk material is described at the meso-scale as a collection of particles. During DEM simulations, a particle itself is merely a rigid body; it is the contact model and model parameters that govern the motion of every individual particle. Before conducting the DEM simulation of concrete flow, the definition of contact model and model parameters is a necessary step. In the literature, different models exist. Some researchers use experimental methods to investigate the aggregate–aggregate and aggregate–boundary contact behaviors, from which new contact laws are proposed [16,30,58,59]). Theoretical derivation also leads to formulas describing the inter-particle interactions due to van der Waals forces or liquid bridging [44,60,61]. The Bingham function is still used frequently while describing the contact behavior [62,63].

To perform DEM simulations, early researchers implemented in-house computer codes; further development leads to open-source software packages such as YADE-OPEN DEM and LIGGGHTS (LAMMPS Improved for General Granular and Granular Heat Transfer Simulations), and commercial programs including UDEC (Universal Distinct Element Code), EDEM software and PFC (Particle Flow Code). In the present research, simulations were conducted using PFC 3D 5.0 (Particle Flow Code 3D Version 5.0) [64]. We used the *linear parallel bond model*, which belongs to the collection of built-in contact models in PFC 5.0 [64,65]. In fact, PFC 5.0 offers nine built-in contact models that are characterized by different model components to be used in different cases. To the authors’ understanding, the other models are less suitable for fresh concrete. For example, the *linear model* mainly describes the contact behavior under compression; therefore, it can be used for dry materials. The *linear contact bond model* has a pair of tensile and shear springs, which, when active, preclude the slip and friction at contact. Other models contain more complex components such as nonlinear spring, nonlinear viscoelastic element, a planar interface, rolling resistance, Kelvin and Maxwell models in series, etc. The fundamental formulations of the linear parallel bond model are briefly provided in the following (details can be found in the software documentation [64]).

**Figure 3:**
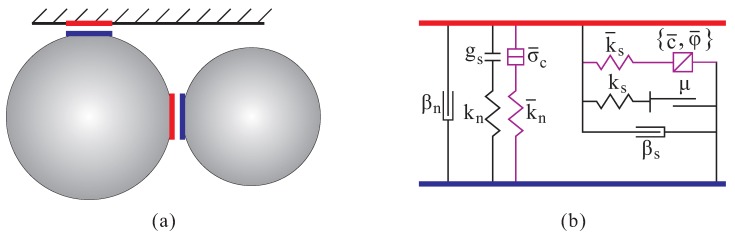
(**a**) Illustration of the contact between particles or between particle and wall; and (**b**) the contact model (the purple colored parallel bond components correspond to the mortar effect).

As illustrated in Figure 3, the contact between two pieces (one of the two pieces must be a particle, the other entity can be either a particle or a wall) consists of a linear contact, a dashpot and a parallel bond component. For fresh concrete, the linear contact mainly controls the overlapping between two pieces (i.e., the behavior under compression), the parallel bond component is associated with the mortar adhesion on the interface (i.e., the behavior under tension), and the dashpot provides numerical stability. When the bond is active, the force and moment at the contact are as follows:

(5) $$F_{c}=F^{l}+F^{d}+\bar{F},\;M_{c}=\bar{M}$$

with

(6) $$F^{l}=-F_{n}^{l}n+F_{s}^{l},\;F^{d}=-F_{n}^{d}n+F_{s}^{d},\;\bar{F}=-{\bar{F}}_{n}n+{\bar{F}}_{s},\;\bar{M}={\bar{M}}_{t}n+{\bar{M}}_{b}$$

where $F^{l}$ is the linear force vector dependent on the spring stiffness $k_{n}$ and $k_{s}$ (in the normal direction n and shear direction s of contact, respectively), the surface gap $g_{s}$, the friction coefficient μ and slip state in shear direction:

(7) $$F_{n}^{l}=\left\{\begin{array}{cc} k_{n}g_{s}, & \mathrm{if}\;g_{s}<0\\ 0, & \mathrm{otherwise} \end{array}\right.;\;F_{s}^{l}=\left\{\begin{array}{cc} F_{s}^{\mathrm{tr}}, & \mathrm{if}\;∥F_{s}^{\mathrm{tr}}∥<-\mu F_{n}^{l}\\ -\mu F_{n}^{l}(F_{s}^{\mathrm{tr}}/∥F_{s}^{\mathrm{tr}}∥), & \mathrm{otherwise} \end{array}\right.$$

with $F_{s}^{\mathrm{tr}}={\left(F_{s}^{l}\right)}_{\mathrm{old}}-k_{s}\Delta \delta_{s}$ the trial shear force dependent on the shear force at the beginning of step and the adjusted incremental relative shear displacement $\Delta \delta_{s}$.

$F^{d}$ is the viscous dashpot force related to the relative velocity at the contact and the damping ratio $\beta_{n}$ and $\beta_{s}$:

(8) $$F_{n}^{d}=\left\{\begin{array}{cc} F^{*}, & \mathrm{full}\mathrm{normal}\\ \min(F^{*},-F_{n}^{l}), & \mathrm{no-tension} \end{array}\right.;\;F_{s}^{d}=\left\{\begin{array}{cc} 2\beta_{s}\sqrt{m_{c}k_{s}}{\dot{\delta }}_{s}, & \mathrm{no}\mathrm{slip}\mathrm{or}\mathrm{full}\mathrm{shear}\\ 0, & \mathrm{slip}\mathrm{and}\mathrm{slip-cut} \end{array}\right.$$

where $F^{*}=2\beta_{n}\sqrt{m_{c}k_{n}}{\dot{\delta }}_{n}$ us the whole dashpot load, $m_{c}$ is the mass of contact-pair, ${\dot{\delta }}_{n}$ is the relative normal velocity and ${\dot{\delta }}_{s}$ is the relative shear velocity vector.

For the parallel bond, the force and moment are calculated as follows

(9) $${\bar{F}}_{n}={\left({\bar{F}}_{n}\right)}_{\mathrm{old}}+{\bar{k}}_{n}\bar{A}\Delta \delta_{n},\;{\bar{F}}_{s}={\left({\bar{F}}_{s}\right)}_{\mathrm{old}}-{\bar{k}}_{s}\bar{A}\Delta \delta_{s}$$

(10) $${\bar{M}}_{t}={\left({\bar{M}}_{t}\right)}_{\mathrm{old}}-{\bar{k}}_{s}\bar{J}\Delta \theta_{t},\;{\bar{M}}_{b}={\left({\bar{M}}_{b}\right)}_{\mathrm{old}}-{\bar{k}}_{n}\bar{I}\Delta \theta_{b}$$

with $\bar{A}$ the cross sectional area of contact, and ${\bar{k}}_{n}$ and ${\bar{k}}_{s}$ the normal and shear stiffnesses (in the form of stress/displacement). ${\bar{M}}_{t}$ is the twisting moment; ${\bar{M}}_{b}$ is the bending moment; $\bar{J}$ and $\bar{I}$ are the twisting and bending moments of inertia, respectively; and $\Delta \theta_{t}$ and $\Delta \theta_{b}$ are the corresponding incremental rotations.

The maximum normal and shear stresses at the contact are calculated as

(11) $$\bar{\sigma }=\frac{{\bar{F}}_{n}}{\bar{A}}+\bar{\beta }\frac{∥{\bar{M}}_{b}∥\bar{R}}{\bar{I}},\;\bar{\tau }=\frac{∥{\bar{F}}_{s}∥}{\bar{A}}+\bar{\beta }\frac{|{\bar{M}}_{t}|\bar{R}}{\bar{J}}$$

with $\bar{\beta }\in \left[0,1\right]$ the moment contribution factor and $\bar{R}$ the radius of contact. If the tensile strength or the shear strength is exceeded:

(12) $$\bar{\sigma }>{\bar{\sigma }}_{c}\;\mathrm{or}\;\bar{\tau }>{\bar{\tau }}_{c}=\bar{c}-\sigma \;\tan\left(\bar{\varphi }\right)$$

the parallel bond breaks.

### 3.3. Material Parameter

As mentioned previously, a DEM model describes the material at the meso-scale; therefore, the defined contact laws and parameters are not equivalent to the conventional macroscopic material property, which can be directly measured with standard experiments. For the determination of mesoscopic model parameter values, a number of methods can be considered. For example, the contact spring stiffness can be derived from the Young’s modulus of material [64]; the frictional coefficient can be measured by laboratory devices [66]; and in [63], empirical formulas are adopted to calculate the contact parameters such as spring constants and dashpot coefficients. In addition, prior to the simulation of concrete flow, a calibration process should always be considered; this process involves running simulation, comparing the results with a standard experiment such as the slump test and inverse analysis, adjusting the parameters and repeating the simulation, until the new parameter values lead to reasonable simulation results (see, e.g., [30,67,68]).

In the present work, a “standard” self-consolidating concrete was assumed, without referring to a particular concrete mix. For the DEM modeling of this virtual SCC, the particles included equal volume of four types of clump (Figure 2), and the grain sizes were in the range of $d_{\mathrm{agg}}\in \left[5,\;20\right]$ mm. The size distribution followed a Gaussian distribution with the mean value 12.5 mm and standard deviation 7.5 mm (note that this approximation should be revised for a specific concrete mix, if the granulometric data are available). The mortar phase was taken into account by means of appropriately defining the particle-particle and particle-boundary contact models. The main parameter values assigned are listed in Table 1. Here, the spring stiffness was indirectly defined by assigning the “effective modulus” $E^{*}$ (and ${\bar{E}}^{*}$), so as to unify the contact property regardless of the particle size. In the table, $\kappa^{*}=k_{n}/k_{s}$ and ${\bar{\kappa }}^{*}={\bar{k}}_{n}/{\bar{k}}_{s}$ control the ratios of normal stiffness to shear stiffness. The coefficient μ considers the friction as well as lubrication due to mortar. The bond strength (${\bar{\sigma }}_{c}$ and $\bar{c}$) corresponds to the yield stress level of mortar.

**Figure 4:**
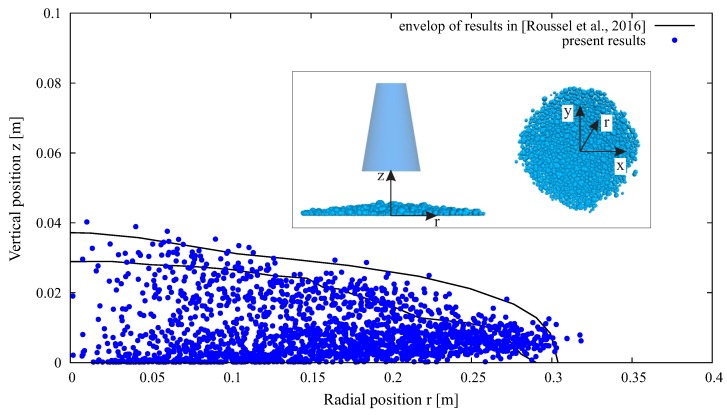
Calibration of DEM model with slump test: comparison between present DEM simulation results (every blue dot indicates the position of a specific aggregate) and the benchmark results in [70].

**Figure 5:**
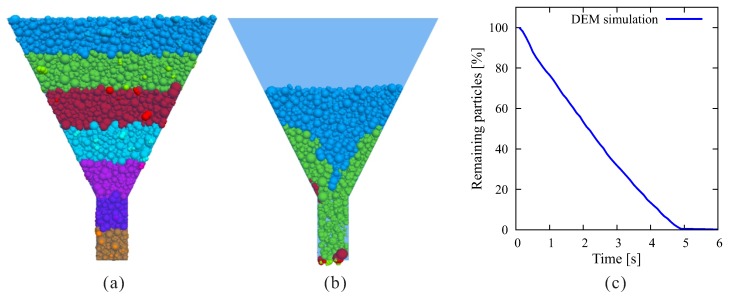
Verification of DEM model with V-funnel test: (**a**) initial state; (**b**) flow status at time = 2.5 s; and (**c**) evolution of remaining particle number.

The parameter values in Table 1 were determined with reference to [56,68,69], and were calibrated according to the slump test benchmark reported in [70]. This was accomplished by “trial and error”: we started with gathering the parameter values from literature; parameters with the same values from different references were generally accepted, and those with different values were tested and adjusted (usually by a scaling factor of 2, 5, or 10), until the slump test benchmark results were well fitted. Figure 4 shows the results of present simulation compared with those obtained from different numerical methods. A further verification example of V-Funnel test [71] was conducted, since the discharge time is an important indicator of the fresh concrete flowability. Experiments on different SCC showed a deviation of discharge time from a few seconds to over ten seconds, despite their highly close slump flow values (see, e.g., the reports by Khayat [72]). Using the parameters listed in Table 1, the discharge time obtained from present DEM simulation was 4.8 s, which reasonably fell within the range from experiments (Figure 5).

## 4. Numerical Simulation of Pumping Behavior

In modern construction projects, fresh concrete is usually provided by mixing plants and pumped through pipelines that can reach a total length of several hundred meters. However, the goal of present work was not the DEM simulation of whole pumping system; instead, we limited our scope to the local simulation of concrete flow through representative pipe units, with the focus on the different performance in various pumping cases characterized by different parameter setting in terms of model geometry, material property and pumping condition. As demonstrated below, the simulation of pumping units can already be used to quantitatively evaluate the pumping performance of whole system. Furthermore, the local simulation results reveal how a specific parameter influences the pumping resistance, and thus provide optimization directions for the pumping program. Nevertheless, the realistic situation in a construction site can be very complicated; there are many influencing factors such as the change of concrete workability, environmental conditions, the assembly of pipeline, the actual pressure and velocity at the outlet of pump. It would be a meaningful and challenging topic to identify all the relevant factors, introduce them appropriately into the numerical model and assess their impact on the pumping performance.

### 4.1. Model Geometry

**Figure 6:**
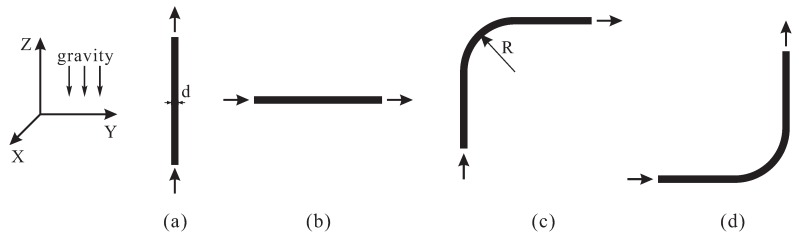
Four representative cases of pipe segment: (**a**) vertical straight pipe; (**b**) horizontal straight pipe; (**c**) upward elbow; and (**d**) downward elbow.

As illustrated in Figure 6, four different cases of pipeline segment were considered, i.e., vertical straight pipe (referred to as “Case A” in the following discussion), horizontal straight pipe (Case B), upward elbow (Case C) and downward elbow (Case D). In general, these units constitute almost the whole pumping pipeline. The pipe had the inner diameter of *d*; the elbow radius was *R*. The geometrical model of pipe was built in CAD software and exported as STL-file, which was later imported by the DEM software to form the boundary (walls).

### 4.2. Material

In the DEM simulations, the reference concrete model was first adopted, as described in Section 3.3. Subsequently, the influence of several parameters such as the aggregate size and shape was investigated by setting different values of particle diameter and clumps with different aspect ratio. In addition, the time dependency of flowability due to, e.g., the hydration and flocculation, were also studied via DEM [68]. Here, before pushing the concrete, we modeled the partially hydrated concrete by randomly selecting the contacts between particles and establishing bonds which were much stiffer than normal.

### 4.3. Simulation Process

**Figure 7:**
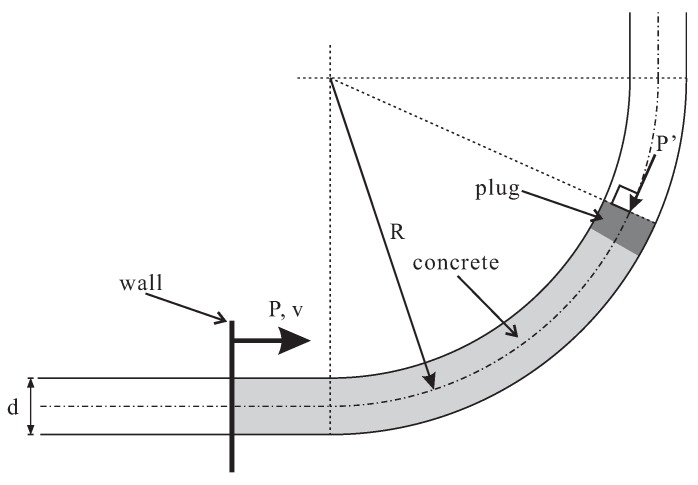
Illustration of pumping conditions.

A unit length (1 m) of fresh concrete was modeled. The flow of fresh concrete was driven by a displacement-controlled wall that was perpendicular to and moved along the pipe axis (Figure 7). A “plug” consisting of a cluster of strongly bonded small balls was generated in the front of concrete. A small pressure $P^{\prime }=1$ kPa was applied to the plug (more specifically, in the form of averaged force acting on every ball and with the direction parallel to the axis of pipeline). This allowed compacting the particles and keeping the shape of concrete unit. Note that $P^{\prime }$ does not correspond to the external pressure; we assumed that the external pressure on this concrete section was balanced out and its effect was neglected; therefore, we focused on the local pumping resistance *P* induced by the gravity, friction and deformation effects.

The pushing of wall consisted of two stages: a first “acceleration stage” in which the velocity of wall was linearly increased until reaching the nominal pumping rate *v*; and the second was the “stable pumping stage” when the wall moved along the pipeline with constant *v*. Since the DEM essentially deald with dynamics, the stability of numerical scheme was ensured by limiting the time step size Δt below a critical value estimated based on the mass and stiffness of particles. Note that PFC offers different options to determine the time resolution. For rigorous tests based on small and simple problems, one may prefer to fix Δt carefully; otherwise, PFC can automatically determine and adapt Δt during simulation. In our simulations, we used the automatic time stepping and it was noticed that Δt was usually in the order of $10^{-6}$ s. The numerical study conducted involved repeatedly executing PFC simulations. Python, a general purpose programming language, is already associated with PFC to allow the manipulation of numerical models via Python scripts. In the present work, short Python codes were written to execute the highly parameterized examples without repeating them by hand.

## 5. Results and Discussions

### 5.1. Influence of Pipe Geometry

#### 5.1.1. Pipe Section

**Figure 8:**
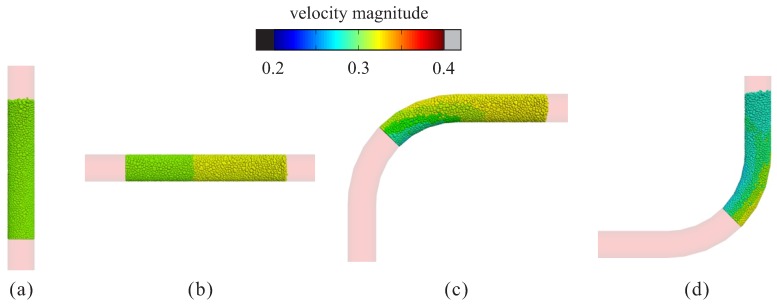
Simulation results of different pipe segments: plot of the velocity magnitude of particles during the stable pumping stage. (**a**) vertical straight pipe; (**b**) horizontal straight pipe; (**c**) upward elbow; and (**d**) downward elbow.

We started with the situations of concrete pumped through different pipe units. As a reference setup, the following condition was considered: pipe diameter d=150 mm, elbow radius R=0.5 m and pumping velocity v=0.3 m/s. In the two figures below, the simulation results of four different pipe units are demonstrated. Figure 8 shows the particles during the stable pumping phase. The color indicates the velocity magnitude, from which different flow behavior can be noticed. In straight pipe sections, the velocity of concrete was nearly uniform (Figure 8a,b); in elbows, the velocity in the outer region was higher than in the inner region (Figure 8c,d).

**Figure 9:**
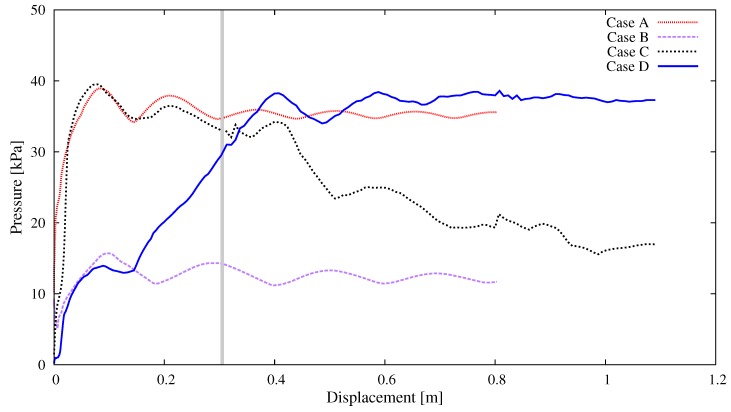
Simulation results of different pipe segments: curves of the thrusting pressure vs. displacement relation.

The thrusting pressure (*P*) vs. displacement (*u*) relationship from each case is plotted in Figure 9. The pressure *P* was calculated as the reaction force on the wall divided by the cross sectional area of pipe, and the displacement *u* was the wall displacement along the axis of pipeline. The vertical gray line at displacement u=0.3 m separated the two stages (i.e., the acceleration stage and stable pumping stage mentioned above). In Case A (vertical pipe), the pressure increased rapidly in the acceleration stage and reached the peak at u=0.08 m, P=38.93 kPa. Afterwards, the curve behaved as a damped oscillation and approached approximately 35 kPa at the end of stable pumping stage. Case B (horizontal pipe) gave a similar curve, yet with much smaller value. The first peak was located at u=0.10 m, P=15.69 kPa and the end value was approximately 12 kPa. Clearly, the difference between these two cases was mainly associated with the gravitational effect. The pumping behavior in Case C (upward elbow) or Case D (downward elbow) was obviously a mixture of Case A and Case B. In Case C, the concrete was first pushed upward through the elbow and then horizontally moved in the pipe; therefore, the pressure first grew quickly in a similar way to Case A and gradually reduced to a level that was slightly higher than Case B. On the contrary, in Case D, the pressure increased slowly first, and stayed at a similar level to that of Case A in the end.

It was observed that the magnitude of pressure loss obtained from DEM simulation corresponded reasonably well to reality. Taking the approximate stable values of 35 kPa for the vertical unit and 12 kPa for the horizontal unit, respectively, the total pressure loss $P^{\mathrm{tot}}$ of the whole pumping system could be approximately estimated:

(13) $$P^{\mathrm{tot}}\approx 35\times L^{\mathrm{ver}}+12\times L^{\mathrm{hor}}$$

with $L^{\mathrm{ver}}$ the pumping height and $L^{\mathrm{hor}}$ the length of horizontal pipeline. For example, for the Burj Khalifa at Dubai, $L^{\mathrm{ver}}=576$ m and $L^{\mathrm{hor}}=83$ m, which leads to $P^{\mathrm{tot}}\approx 21.15$ MPa; that is 23% higher than the reported value 17.1 MPa [15]. For the Shanghai Tower, $L^{\mathrm{ver}}=582$ m and $L^{\mathrm{hor}}=150$ m, which gives $P^{\mathrm{tot}}\approx 22.17$ MPa; that is 5% smaller than the recorded 23.4 MPa [73]. These results, although obtained from simple calculation, quantitatively support the plausibility of present DEM simulation and demonstrate the potential of using DEM analysis as the foundation of full analysis of pumping problems in the future.

From the diagram, it is also interesting to see that, contrary to the common idea that the pressure loss induced by elbows should be significantly larger than straight pipe, the pumping resistance did not differ much. This observation agrees with the finding in [13]; however, it should be emphasized that it only applies to the situation of relatively smooth pumping. Unfavorable cases occur usually at the elbow.

In the following parametric study, Case D (Figure 8d) was selected as the reference situation, where pipe diameter d=150 mm, elbow radius R=0.5 m, pumping velocity v=0.3 m/s, and the “standard” concrete described in Section 3.3 was modeled. Six groups of numerical examples were conducted. Every group contained several cases that focused on one of the following six parameters: pipe diameter, pipe curvature, aggregate size, aggregate shape, pumping velocity and time dependency, by means of changing only the value of selected parameter.

#### 5.1.2. Pipe Diameter

**Figure 10:**
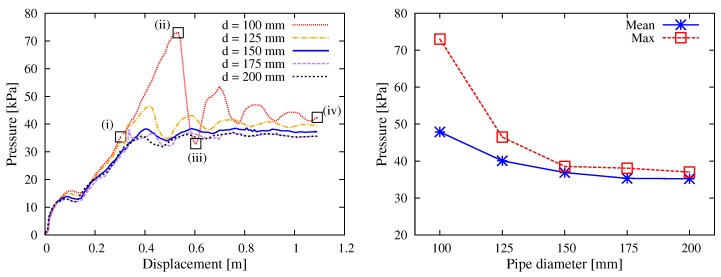
Simulation results with respect to the pipe diameter: (**left**) curves of the thrusting pressure vs. displacement relationship; and (**right**) mean and max values during the stable pumping stage.

It is common sense that a larger size of pipe cross section not only allows raising the pumping efficiency but also reduces the risk of clogging. Considering the reality and for the purpose of comparison, the value of inner diameter was assumed to be d∈{100,125,150,175,200} mm. As the simulation results show, the smaller the pipe diameter was, the higher resistance the pumping confronted (Figure 10). While there was only small difference among the pressure levels for the cases of diameter d= 150 mm, 175 mm and 200 mm, a diameter that was smaller than 150 mm clearly led to a nonlinear increase of pressure. The comparison of mean values already showed this tendency; more remarkably, the maximum pressure in the case of d=100 mm was found to be 72.99 kPa, which was nearly double (189%) that of d=150 mm (38.58 kPa). The simulations coincided with the choice of d=150 mm in many construction projects.

**Figure 11:**
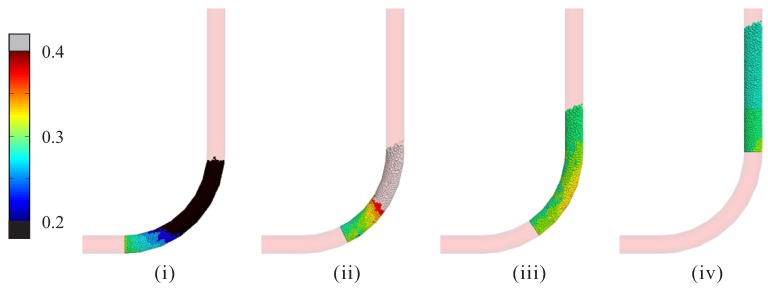
Simulation results with respect to the pipe diameter: plot of the velocity magnitude of particles at different states during the stable pumping stage for the case of d=100 mm.

For the result of d=100 mm in Figure 10, the appearance of oscillation of pressure level did not indicate any structural vibration; in fact, it was a result of repeated shoving and relaxing of particles, since smaller pipes restricted the space for the lateral motion of aggregates and increased the probability of interlocking among them. Figure 11 illustrates the particle velocity at different states (i, ii, iii and iv), as marked in Figure 10. As can be seen, at the beginning of stable pumping stage (i), while the end of concrete section was pushed with a constant velocity (v=0.3 m/s), the forepart clearly moved more slowly (v<0.2 m/s) and became a hindrance to this concrete section; this led to continuous ascending of the thrusting pressure. With a short period of pushing, the forepart gained sufficient velocity to release the hindrance (ii), and the reaction force on the wall started to drop rapidly (iii). After repeated campaign between the motion and resistance, at the end of simulation, the concrete reached a relatively uniform velocity distribution and the oscillation of curve became much smaller (iv).

#### 5.1.3. Elbow Curvature

**Figure 12:**
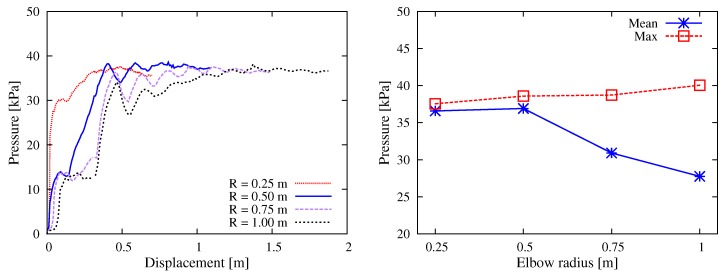
Simulation results with respect to the elbow curvature: (**left**) curves of the thrusting pressure vs. displacement relationship; and (**right**) mean and max values during the stable pumping stage.

It is usually believed that large curvature of pipeline raises the risk of clogging during concrete pumping. In practice, elbows with *R* = 0.5–1.0 m are frequently used. The present parametric study investigated four cases of elbow radius R∈{0.25,0.5,0.75,1.0} m. As shown in Figure 12, larger *R* (smaller curvature) reduced the pumping difficulty marginally. (Note that the simulation stopped when the wall rotated by $90^{\circ }$ and all particles left the elbow; consequently, the total thrusting displacement was different.) The pressure-displacement curves showed that, in general, the maximum values of pressure were nearly the same; nevertheless, smaller curvature allowed relatively mild ascending of pressure, leading to smaller mean value of pressure. The simulation suggested that the choice of elbow curvature did not affect the pumping performance critically; however, where appropriate, elbows with smaller curvature are still recommended.

### 5.2. Influence of Material

#### 5.2.1. Aggregate Size

**Figure 13:**
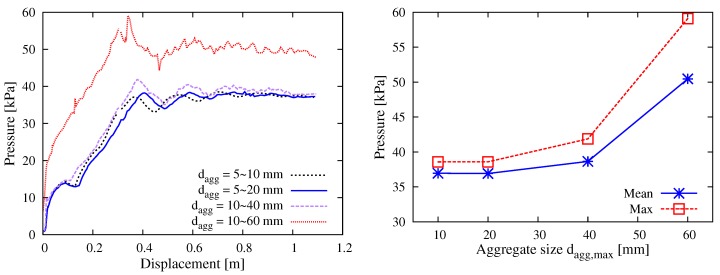
Simulation results with respect to the aggregate size: (**left**) curves of the thrusting pressure vs. displacement relationship; and (**right**) mean and max values during the stable pumping stage.

Engineering experience tells that, qualitatively, large aggregates reduce the flowability of fresh concrete and increase the pumping risks. To obtain optimal pumpability, the maximum aggregate size considered for the self-consolidating concrete is often limited to 20 mm. The present study agreed well with the practical experience and further provided a quantitative basis for understanding the influence of aggregate size on the pumping behavior. As shown in Figure 13, four cases of DEM simulation characterized by the particle size $d_{\mathrm{agg}}$ ranging 5–10 mm, 5–20 mm, 10–40 mm and 10–60 mm were conduced. The results reveal that the pressure levels for the cases of $d_{\mathrm{agg}}$ = 5–10 mm and $d_{\mathrm{agg}}$ = 5–20 mm were almost the same; with the growth of aggregate size, the pumping pressure (both mean and max values) showed a nonlinear tendency of increase; in particular, if the aggregate sizes were 10–60 mm, the raise of pressure level became remarkable.

#### 5.2.2. Aggregate Shape

**Figure 14:**
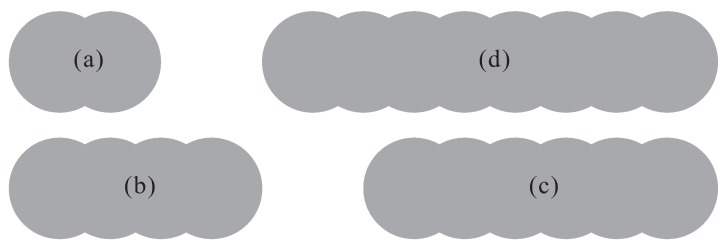
Clump templates used to create concrete aggregates with shape factors equal to: (**a**) 1.5; (**b**) 2.5; (**c**) 3.5; and (**d**) 4.5.

**Figure 15:**
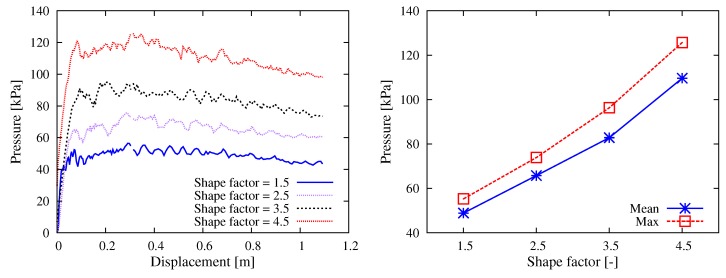
Simulation results with respect to the aggregate shape: (**left**) curves of the thrusting pressure vs. displacement relationship; and (**right**) mean and max values during the stable pumping stage.

For the smooth pumping of SCC, the concrete mix design requires that the aggregates be “well-shaped”, which means the closer to spherical shape the better. Here, we quantitatively studied the impact of aggregate shape factor on the pumping resistance. As sketched in Figure 14, four different values (1.5, 2.5, 3.5 and 4.5) of aggregate shape factor were considered by creating clump templates. In each DEM simulation, only one template was used to generate all the particles. Figure 15 contains the simulation results regarding the shape factor of aggregates. It was quite clear that poorly shaped aggregates with large value of shape factor caused high pumping resistance. In addition, it was noticed that the stress level showed a linear dependency on the value of shape factor.

### 5.3. Influence of Pumping Condition

#### 5.3.1. Pumping Velocity

**Figure 16:**
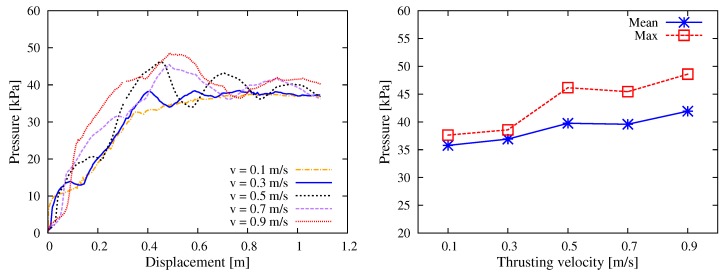
Simulation results with respect to the pumping velocity: (**left**) curves of the thrusting pressure vs. displacement relationship; and (**right**) mean and max values during the stable pumping stage.

The thrusting velocity *v* in the stable pumping stage is usually in the order of $10^{-1}$ m/s. For example, during the construction of the world’s tallest building, Burj Khalifa, it is reported in [15] that the flow rate was 21.3 m${}^{3}$/h (which is converted to the average flow velocity in pipe as $v_{0}=0.33$ m/s). To study the influence of velocity, different values of v∈{0.1,0.3,0.5,0.7,0.9} m/s were tested here. Figure 16 shows the results of pumping reaction, from which one can see that in general the pumping pressure increased more or less in a linear manner with respect to the thrusting velocity. This finding agrees with a number of experimental and analytical results that the pumping pressure linearly depends on the flow rate (e.g., [13,14,15]).

#### 5.3.2. Time Dependency

In practice, it is required to pump the concrete within a short time (e.g., 1 h) after the concrete is freshly produced at the mixing plant; otherwise, the workability of concrete deteriorates with time due to several reasons such as hydration. Here, we conducted the DEM simulation to test different hydration states, without referring to any exact value in reality. We randomly selected a proportion of particle contacts and assigned higher values to the bond stiffness ${\bar{E}}^{*}$. For the present testing purpose, the proportion of “hydrated” contacts was assumed in the range of {0%,5%,10%,15%,20%}; the stiffness was given the value ${\bar{E}}^{*}\in \left\{2.0\times 10^{4},\;1.0\times 10^{6},\;1.0\times 10^{7},\;1.0\times 10^{8},\;1.0\times 10^{9}\right\}$ Pa. In these examples, the strength of hydrated bond was set as ${\bar{\sigma }}_{c}=1.0\times 10^{6}$ Pa, $\bar{c}=5.0\times 10^{5}$ Pa.

**Figure 17:**
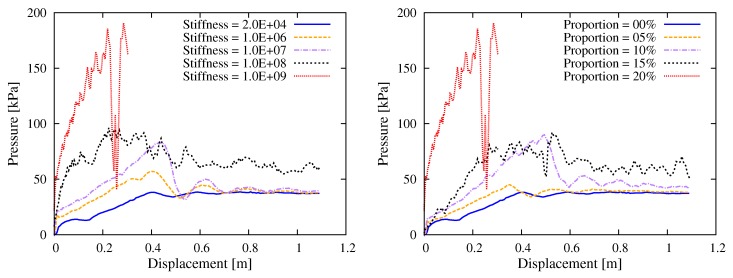
Simulation results with respect to the hydration degree: (**left**) curves of the thrusting pressure vs. displacement relationship for different values of hydrated bond stiffness; and (**right**) curves of the thrusting pressure vs. displacement relationship for different proportions of hydrated contact.

Figure 17 shows the results by fixing the hydration proportion as 20% and changing the bond stiffness (Figure 17, left), and by fixing the bond stiffness as ${\bar{E}}^{*}=1.0\times 10^{9}$ Pa, but with different hydration proportions (Figure 17, right). These two diagrams clearly indicate the increase of pumping resistance with respect to either the bonding stiffness or the hydration ratio.

**Figure 18:**
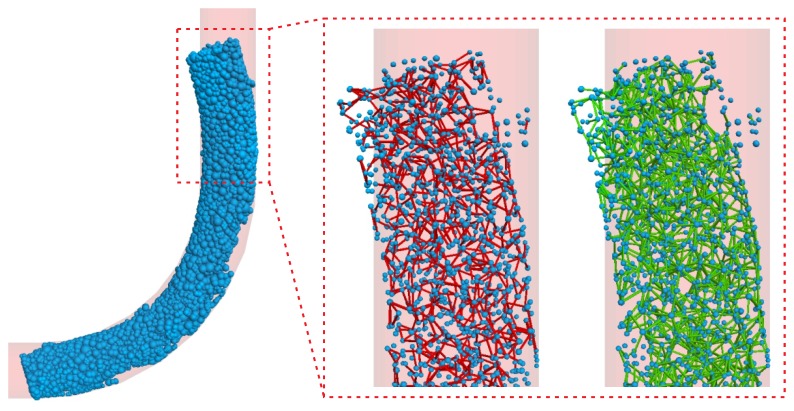
Simulation results with respect to hydration: The excessive case of 20% contacts hydrated with ${\bar{E}}^{*}=1.0\times 10^{9}$.

It was interesting to look into the extreme situation of ${\bar{E}}^{*}=1.0\times 10^{9}$ and hydration proportion equaling to 20% (Figure 18). Clearly, if 20% of all the contacts were highly hydrated, this concrete section became almost entirely hardened. (One may notice that the head of concrete arc extruded the pipe; this was due to the relatively small particle-wall contact stiffness, which was kept with intention of illustration.) The right part of figure shows the detail of contacts. The red lines indicate the 2109 hydrated contacts that remained unchanged, while the green lines represent the residual 3997 unhydrated contacts out of the initial 8094 contacts before pumping.

## 6. Concluding Remarks

The present paper reports the DEM modeling and simulation of the pumping behavior of self-consolidating concrete at a local level, considering different situations of concrete pushed through a pipeline unit. The flow of concrete is reflected by the motion of particles that represent coarse aggregates, and the mortar phase is physically taken into account as the contact behavior. A series of parametric studies was carried out, with respect to the influence of pipe geometry, material property and pumping condition. The simulation results suggest that the gravitational effect takes a big part of the pumping resistance; an elbow does not cause significant increase of pressure level; smaller pipe diameter or larger aggregate size leads to dramatic pressure growth in a nonlinear manner with increasing slope; the aggregate shape factor or the pumping rate has a linear influence on the pumping resistance; and the hydration of fresh concrete should be controlled. In general, the DEM simulation of concrete flow not only helps to understand the details of pumping behavior, but also inspires new concrete pumping techniques that can be considered to eliminate pumping risks and to confront more challenging circumstances such as record-breaking pumping height in the future.

Limitations have also been noticed during the present numerical study, from which subsequent research is being considered. The mesoscopic model of fresh concrete should be improved, including more accurate geometrical representation of aggregates [31], more sophisticated contact laws and substantial investigation of the inter-phase mechanisms and model parameters [61], such that the major mechanisms involved at the mesoscale can be adequately captured. At the local level, the DEM simulation should be further refined, considering, e.g., the influence of fluid phase, possibly by using coupled DEM/CFD analyses. It would also be meaningful to compare the DEM-based modeling with the detailed assessment of local phenomena such as secondary flow in the vicinity of wall and complex trajectories of particles, using high-fidelity CFD analysis [74,75]. Finally, the approach from numerical simulation to the accurate and fast prediction of system pumpability should be developed to serve realistic construction projects directly.

## Figures and Tables

**Table 1:** Main parameter values considered for the in the present DEM simulation.

Parameter	Symbol	Value	Unit
aggregate density	ρ	$2.5\times 10^{3}$	[kg/m${}^{3}$]
aggregate size	$d^{\mathrm{agg}}$	$5\times 10^{-3}∼20\times 10^{-3}$	[m]
linear contact effective modulus	$E^{*}$	$1.0\times 10^{5}$	[N/m${}^{2}$]
linear contact normal-to-shear stiffness ratio	$\kappa^{*}$	2.0	[-]
linear contact friction coefficient	μ	0.05	[-]
normal critical damping ratio	$\beta_{n}$	0.5	[-]
shear critical damping ratio	$\beta_{s}$	0.0	[-]
parallel bond effective modulus	${\bar{E}}^{*}$	$2.0\times 10^{4}$	[N/m${}^{2}$]
parallel bond normal-to-shear stiffness ratio	${\bar{\kappa }}^{*}$	2.0	[-]
parallel bond tensile strength	${\bar{\sigma }}_{c}$	10.0	[N/m${}^{2}$]
parallel bond cohesion	$\bar{c}$	5.0	[N/m${}^{2}$]
parallel bond friction angle	$\bar{\varphi }$	30	[${}^{\circ }$]
